# Binge Drinking Associations with Patrons’ Risk Behaviors and Alcohol Effects after Leaving a Nightclub: Sex Differences in the "*Balada com Ciência*" Portal Survey Study in Brazil

**DOI:** 10.1371/journal.pone.0133646

**Published:** 2015-08-19

**Authors:** Zila M. Sanchez, Karen J. Ribeiro, Gabriela A. Wagner

**Affiliations:** Department of Preventive Medicine, Universidade Federal de São Paulo, São Paulo, Brazil; Universidade de São Paulo, BRAZIL

## Abstract

The purpose of the present study was to investigate the potential associations of binge drinking detected at the exit of nightclubs and risk behaviors and alcohol effects just after leaving the venue in a representative sample of Brazilian nightclub patrons according to sex. For this purpose, a portal survey study called *Balada com Ciência* was conducted in 2013 in the megacity of São Paulo, Brazil, using a two-stage cluster sampling survey design. Individual-level data were collected in 2422 subjects at the entrance and 1822 subjects at the exit of 31 nightclubs, and breath alcohol concentration (BrAC) was measured using a breathalyzer. The following day, 1222 patrons answered an online follow-up survey that included questions about risk behaviors and alcohol effects practiced just after leaving the nightclub. Weighted logistic regressions were used to analyze binge drinking associated with risk behaviors by sex. For both sexes, the most prevalent risk behaviors practiced after leaving a nightclub were drinking and driving (men=27.9%; women=20.4%), the use of illicit drugs (men=15.8%; women=9.4%) and risky sexual behavior (men=11.4%; women=6.8%). The practice of binge drinking increased the behavior of illicit drug use after leaving the nightclub by 2.54 times [95% CI: 1.26-5.09] among men who drank and increased the risk of an episode of new alcohol use by 5.80 times [95% CI: 1.50-22.44] among women who drank. Alcoholic blackouts were more prevalent among men [OR=8.92; 95% CI: 3.83-20.80] and women [OR= 5.31; 95% CI: 1.68-16.84] whose BrAC was equivalent to binge drinking compared with patrons with a lower BrAC. Public policies aiming to reduce patrons’ BrAC at the exit of nightclubs, such as staff training in responsible beverage service and legislation to prevent alcohol sales to drunk individuals, would be useful to protect patrons from the risk behaviors associated with binge drinking in nightclubs.

## Introduction

Alcohol consumption is a well-known cause of morbidity, mortality and social damage around the world and is a major component of the global burden of disease, particularly in the Americas and Europe [1, 2]. Compared with the other most populous countries, Brazil is ranked as having the second highest rate of major complications resulting from alcohol consumption, according to disability-adjusted life years lost [3].

Noteworthy is the fact that the severity of the consequences of alcohol consumption depends on the frequency of consumption and the quantities consumed [4]. A standard pattern of risky consumption that has aroused international interest and only recently began to be investigated in Brazil is called "binge drinking" (BD) [5] or "heavy episodic drinking" [6]. This pattern is usually characterized by the use of at least four doses of alcohol on a single occasion for women and five doses for men, which leads to an ethanol concentration in the blood of 0.08% or higher [7]. The regulation of the four drink cutoff for women was defined based on women’s lower rate of metabolism for alcohol, which leads to higher blood alcohol levels compared with men for the same quantity of alcohol [8]. However, the BD definition is controversial and permeated by the conceptualization of conflicts, which are influenced by the culture of the use and pharmacokinetic aspects of alcohol [9, 10]. These episodes of acute alcohol abuse not only have an influence on overall mortality but also contribute to acute consequences, particularly accidents [11] and violence [12], thus endangering the intoxicated and the community. BD is associated with higher rates of sexual abuse, suicide attempts, unprotected sex, unwanted pregnancies, alcohol overdose, falls, gastritis and pancreatitis [13].

However, it has been emphasized that there are clear gender differences regarding the effects of alcohol [14]. Men are consistently more than twice as likely as women to report episodes of alcohol intoxication and alcoholism [15–19]. However, after fewer years of heavy drinking, alcoholic women are more likely than men to develop cirrhosis [20], alcohol-induced damage of the heart muscle [21] and nerve damage [22]. Finally, the metabolism of alcohol is affected by the body’s amount of water and fat, which are responsible for the difference in the number of doses that define an episode of binge drinking for men and women [14].

Binge drinking occurs mostly in recreational settings, such as nightclubs [23]. Nightclubs are places attended mostly by youth and young adults that seek different forms of entertainment at these venues, in which the use of alcohol and other drugs act as important mediators [24]. However, they are also places where breaking social rules is tolerated and pleasure is stimulated [25], which contributes to a higher exposure of patrons to risks [26]. International findings show that the excessive consumption of alcohol in nightclubs and bars is associated with more episodes of physical aggression [27], sexual risk behaviors [28] and sexual violence [29] in these establishments and traffic accidents [30] on the way to or back from the venue.

In Brazil, one study reported that 40% of young people (18–24 years) engaged in BD at least once in the 12 months preceding the survey, and nightclubs are the places of choice for this practice [31]. Among teenage high school students in this country, the picture is even more alarming, with 35% of high school students in one study reporting engaging in BD in the month preceding the survey; again, nightclubs are the location of choice for this practice [32].

It is noteworthy that most of the literature concerning the consequences of binge drinking in nightclubs is restricted to risky behaviors that occur within the venue. However, considering that patrons leave the venues with high alcoholic concentrations [33], the consequences of alcoholic intoxication can also be evaluated and noted in the behavior practiced just after leaving the establishment, offering important results for the development of public policies. Thus, the objectives of this paper were 1) to describe the frequency of risk behaviors practiced by nightclub patrons just after the departure from the venues and 2) to identify possible associations between the occurrence of these events and of alcohol effects with the standard binge drinking values (breath alcohol concentration ≥ 0.38 mg/L) measured on the breath of this population when exiting these establishments, according to the sex of the patrons.

## Materials and Methods

### Sampling

This study was a two-stage cluster sampling portal survey among nightclub patrons interviewed at the entrance and exit of nightclubs and the following day. The first stage consisted of a systematic sample of nightclubs with a selection probability proportional to the nightclub’s maximum capacity. The second stage was a systematic sampling of every third person in the entrance line of the nightclubs. Data were collected during the first semester of 2013 in the city of São Paulo, Brazil.

For the selection of the venues, nightclubs were defined as leisure venues that sell alcoholic beverages, have one or more dance floors, and offer individual control of patron entry and exit through the payment of an entrance fee. The nightclub frame list was created by an active search of magazines and guides specializing in leisure activities and a search of the first ten pages returned from a Google search using the following key words: ‘São Paulo bars, nightclubs and discos’ (in Portuguese). The final frame list consisted of 150 nightclubs meeting the inclusion criteria, from which 40 nightclubs and potential replacements were chosen [34].

A sample size of 1600 patrons was calculated so that the prevalence of alcohol intoxication could be estimated to within 5 percentage points (absolute precision) of the true value set to 50% (maximum variance) with 95% confidence with two stages of cluster sampling and a design effect of 2 [35]. Taking into account a refusal rate of 30% and a maximum follow-up loss of 40% from patron entrance to patron exit, which was based on previous studies by Clapp et al. [36], it was determined that 2912 patrons should be initially approached. The adopted inclusion criteria were the intention to enter the nightclub and being at least 18 years old. In the case of refusal, data on age and sex were registered, and the next person in line was approached.

Details on the sample weights calculated from non-response and post-stratification are described in Carlini et al. [37].

### Data collection and instruments

The selected patrons who agreed to participate in the study answered a questionnaire on sociodemographic variables, the practice of pre-drinking, alcohol use patterns, drug use, and other risk behaviors in nightclubs in the past 12 months prior to the interview. The patrons also had their breath alcohol concentrations (BrAC) measured at the time of the interview by means of a breathalyzer (calibrated Draguer Alcoltest 7410 plus RS), and each patron received a bracelet with a unique numeric code for identification at the time of nightclub exit. At the nightclub exit line, the same participating patrons—identified by their bracelets—were approached once more and invited to answer another questionnaire regarding the use of alcohol, illicit drugs and other risk behaviors they could have engaged in while inside the nightclub. At the end of the exit interview, breath alcohol concentration was measured once more. Additionally, a project folder containing information regarding the post-nightclub questionnaire that would be sent by e-mail the next day was handed to the participants.

On the day after the nightclub interview, the link to the online post-nightclub questionnaire was sent by e-mail to the interviewees who had answered the questionnaire in the entrance line of the nightclub 12 hours before. The first module consisted of questions regarding patron risk behaviors after exiting the nightclub, and the second module consisted of questions from the AUDIT, which served as a basis for the identification of high-risk groups and their randomization to participate in an electronic social norms intervention to reduce harmful drinking.

**Fig 1:**
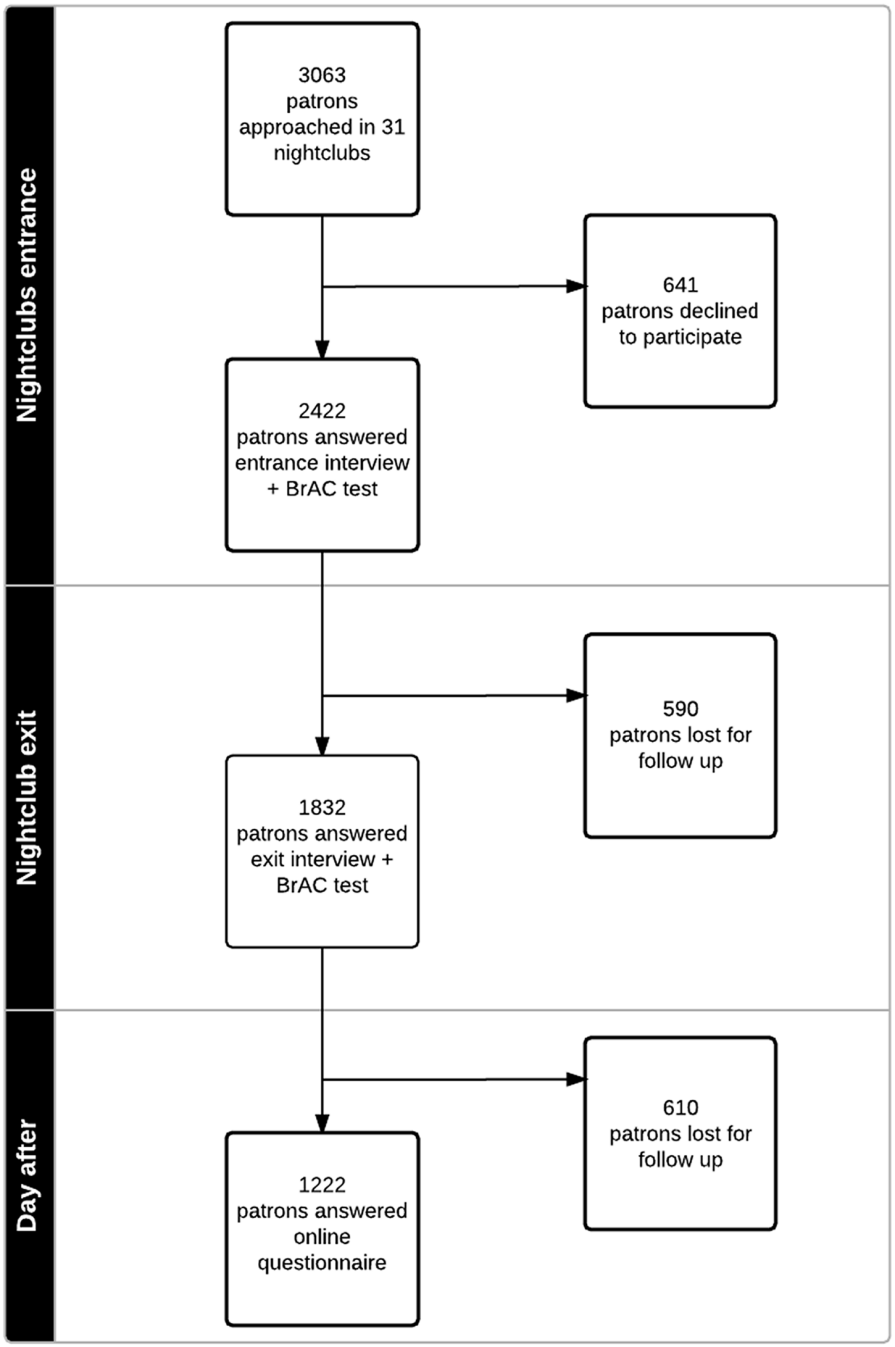
Flowchart for patron’s recruitment and data collection at three time points, “Balada com Ciência” Portal Survey, São Paulo, Brazil, 2013.

This manuscript presents the results for the first module of the online survey “behaviors after leaving the nightclub”, as dependent variables and the data from the exit BrAC (mg/L) and entrance interviews as independent variables. Fig 1 describes the flow of participants in the interviews of the study.

### Variables

#### Dependent variables

To proceed with the analysis, the 40 binary (yes/no) questions about risk behaviors were grouped to create eight variables. Data on risk behaviors and alcohol effects were obtained from the interviewees’ responses to the online follow-up questionnaire. Six categories of risk behaviors were created: “drink and drive”; “illicit drug use after leaving”; “new alcohol use”; “violent behavior”; “accidents”; “sexual risk behavior”; and “policy involvement”. Two categories of alcohol effects identified after leaving the nightclub were created: “physical complications” and "blackout". All these variables were categorized as “no” or “yes”.

The variable “illicit drug use after leaving” included the use of at least one of the following substances: marijuana or hashish, cocaine, ecstasy, tobacco, crack, inhalants, ketamine, methamphetamine, other amphetamines, benzodiazepines or hallucinogens (such as LSD, mushrooms and peyote). The variable "violent behavior" included self-reported fights or arguments with family, fights or discussions with friends, colleagues or boyfriend/girlfriend, or fights or arguments with strangers, and the suffering or practicing of physical assault and vandalism (e.g., stealing traffic signs, plundering). "Accidents" included "I suffered a car accident" and "I suffered some other accident". The variable "sexual risk behavior" included the occurrence of sexual intercourse without a condom with a steady partner, sexual intercourse without a condom with a casual partner, the occurrence of sexual intercourse in which there was regret and sexual intercourse against their will. “Physical complications” included “vomited; felt physically bad; got sick” and “fainted”. The variable "blackout" included little or no memories of the period. When scoring any of these topics, the variable under which it was located was considered a case.

#### Independent variables

The following socio-demographic variables were also analyzed: age (18–24, 25–34, 35–44, 45+); marital status (single, married, others); and education (middle school, high school, college, graduation). Socioeconomic status (SES) was evaluated as indexed in relation to a highly standardized Brazilian survey assessment of SES known as the Associação Brasileira de Empresas de Pesquisa (Brazilian Association of Research Agencies) index. This index is based on the education level of the head of the household, the possession of various types of household goods (e.g., television sets) and the number of housekeepers. This scale was used to sort participants into standardized subgroups labeled A–E (in which A was the highest economic strata). To facilitate the interpretation and improve the accuracy of estimates in the regression models, for the SES variable, the D and E classes were grouped together [38].

BrAC was measured with a breathalyzer test after each interview and categorized in the analysis into three groups: “non-drinker”, “BrAC 0.01–0.37 mg/L”, or “BrAC ≥0.38 mg/L”. Binge drinking was defined as a BrAC ≥0.38 mg/l, which corresponds to a blood alcohol concentration of 0.08% [(mean concentration for a binge drinking episode) [7, 39]. Except for the measure of BrAC, all other variables were self-reported.

BrAC is the main independent variable and risky behaviors are dependent variables in each logistic regression because binge drinking occurred before the risk behavior or alcohol effect (there is a temporal relationship between these variables.

**Fig 2:**
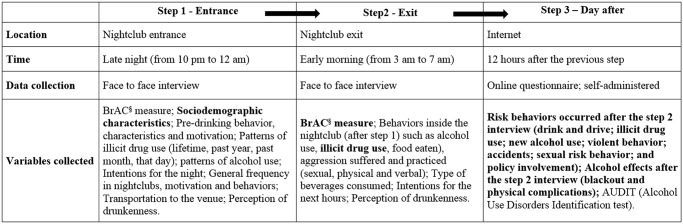
Description of the 3 time points of data collection among nightclub patrons in the study “Balada com Ciência” Portal Survey, São Paulo, Brazil, 2013. ^**§**^BrAC—Breath alcohol concentration; Only the bold variables were used in the present analysis.

Fig 2 describes the moment in which each of the variables used in the statistical analysis were collected and brings the variables used in the analysis of this manuscript (see S1 File).

### Statistical analysis

A descriptive analysis was conducted of the socio-demographic characteristics, BrAC measured at the exit of the nightclub, illicit drug use and risk behaviors according to sex. All the data are presented as proportions. The distribution of BrAC measured at the exit of the nightclub according to socio-demographic, illicit drug use and risk behavior characteristics was analyzed. Intergroup comparisons were performed using Pearson’s chi-square (χ^2^) test with the Rao-Scott correction [40]. The null hypothesis was rejected at a level of significance of 5%. The association between each risk behavior and BrAC was analyzed using logistic regression models stratified by sex, with a backward stepwise procedure. The reference categories for this analysis were “no driving”, “no illicit drug use”, “no new alcohol use”, “no violence behavior”, “no physical complication”, “no sexual risk behavior”, “no sexual assault”, “no physical discomfort”, “no policy involvement” and “no blackout”. To evaluate the possible effects of binge drinking BrAC measures at the exit of the nightclub on the occurrence of these risk behaviors just after leaving the venue, the models considered only drinkers (BrAC≥0.01 mg/L). The models were adjusted by age, marital status, education, socioeconomic status and illicit drug use (use of drugs concomitantly to alcohol use inside the nightclub: marijuana or hashish, cocaine, ecstasy, tobacco, crack, inhalants, ketamine, methamphetamine, other amphetamines, benzodiazepines or hallucinogens). These analyses were performed using the Stata statistical software package, version 13. In this model, the magnitude of the associations was estimated using odds ratios and their respective 95% CIs. The analysis incorporated weights to correct for the different selection probabilities of the participants, and the results are expressed as weighted values.

### Ethical considerations

The Research Ethics Committee of the Universidade Federal de São Paulo (protocol 21477) approved this study. No interviews were conducted with patrons showing signs of severe intoxication, following the guidelines for screening described in Perham et al. [41].

## Results

A total of 1222 individuals answered the 3 phases of the study (interview at the entrance and exit of the nightclub and the online questionnaire the following day). At the time of the interviews, the population of those surveyed corresponded to 5789 nightclub patrons of the city of São Paulo, Brazil. These patrons were primarily young (18–24 years old), single, middle to high social class and with high-school education completed for both sexes. Binge drinking BrAC levels at the exit were detected among 29.6% of the men and 22.1% of the women interviewed. As for risk behaviors, despite the low frequency of occurrence, men were usually exposed to a greater extent than women. The most prevalent risk behavior after leaving the nightclub for both sexes was drinking and driving (Table 1).

**Table 1:** Weighted distribution of sociodemographic characteristics, binge drinking, risk behaviors and alcohol effects after leaving a nightclub by sex among patrons in São Paulo, Brazil–“*Balada com Ciência*” portal survey (N = 1222).

			MEN			WOMEN			
			N = 694			N = 528			
	Variables	Categories	Wg%^#^	Wgn^&^	Unwgn^$^	Wg%^#^	Wgn^&^	Unwgn^$^	p-value*
Sociodemographics	Age	< = 24	48.8	1488	344	52.0	1426	281	0.355
		25–34	36.8	1122	256	36.6	1003	182	
		35–44	9.9	301	68	10.6	290	60	
		45+	4.5	137	26	0.8	23	5	
	Marital status	Single	89.7	2757	632	86.5	2395	458	0.175
		Married	6.5	181	43	8.1	227	47	
		Other	3.8	103	17	5.4	112	21	
	Education	Middle school	2.5	26	19	2.9	12	17	0.508
		High school	52.3	603	361	55.8	480	295	
		College	36.0	2088	239	30.1	1950	159	
		Graduation	9.2	276	64	10.4	284	54	
	Socioeconomic Status^¥^	A	28.7	876	193	26.6	731	135	0.362
		B	55.8	1700	381	58.1	1592	311	
		C	14.3	440	114	14.0	385	74	
		D/E	1.1	32	6	1.3	34	8	
Inside the nightclub	Binge drinking^§^	Non-drinker	41.5	1264	292	55.3	1514	285	0.076
		BrAC 0.01–0.37 mg/L	28.9	880	197	22.6	618	124	
		BrAC≥0.38 mg/L	29.6	903	205	22.1	604	118	
	Illicit drug use inside nightclub	Yes	32.9	1002	226	22.1	605	116	0.002
After leaving the nightclub	Illicit drug use after leaving	Yes	15.8	481	108	9.4	257	49	0.001
	Drink and drive	Yes	27.9	850	194	20.4	558	98	0.032
	New alcohol use	Yes	9.2	281	67	7.1	194	37	0.221
	Violent behavior	Yes	3.8	117	33	2.8	78	16	0.358
	Accidents	Yes	1.0	29	8	0.7	18	3	0.597
	Sexual risk behavior	Yes	11.4	81	346	6.8	36	186	0.003
	Policy involvement	Yes	0.4	13	4	0.8	21	4	0.363
	Physical complications	Yes	7.4	224	50	9.4	257	43	0.432
	Blackout	Yes	9.7	297	72	8.1	223	40	0.507

^#^ Weighted proportions in percentage;

^&^ Weighted sample size (data were weighted to be representative of the nightclubs in São Paulo, Brazil);

^$^ Sample size.

^¥^ Socioeconomic status classification by ABEP (ABEP, 2012).

* Rao-Scott chi-square test.

^**§**^ BrAC—Breath alcohol concentration.

Among the individuals who drank in the nightclubs, the sociodemographic characteristics, illicit drug use inside the venue, risk behaviors after leaving the nightclub and alcohol effects were compared according to the alcohol concentration in their breath at the exit and by sex, as shown in Tables 2 and 3. There were no sociodemographic differences in each alcoholic group (binge vs. non-binge) for both sexes. However, a trend of significance (p = 0.052) was noted for illicit drug use within the nightclub among women, i.e., women who presented an alcoholic dosage equivalent to the practice of binge drinking reported lower consumption of illegal drugs within the venue compared with women who drank but had lower BrAC measures (Table 2). Regarding the risk behaviors practiced after leaving the nightclub and the possible effects of alcohol, there was a higher proportion of illicit drug use after leaving, physical complications and blackout among men with a BrAC ≥0.38 mg/L compared with drinkers with lower alcoholic strengths. Among women, there was a higher prevalence of new episodes of alcohol use and blackout after leaving the nightclub among those with a BrAC ≥0.38 mg/L (Table 3).

**Table 2:** Weighted distribution of sociodemographic and drug use characteristics according to sex and BrAC measured at nightclub exits among patrons in São Paulo, Brazil–“*Balada com Ciência*” portal survey (N = 664 drinkers).

		MEN			WOMEN		
		BrAC^§^ 0.01–0.37 mg/L	BrAC≥0.38 mg/L		BrAC 0.01–0.37 mg/L	BrAC≥0.38 mg/L	
		(no binge drinking)	(binge drinking)		(no binge drinking)	(binge drinking)	
Variables	Categories	(N = 197)	(N = 205)	p-value*	(N = 124)	(N = 116)	p-value*
Age	< = 24	52.9	54.4	0.878	56.4	62.2	0.454
	25–34	34.6	35.2		38.2	30.8	
	35–44	8.7	8.3		5.5	6.0	
	45+	3.8	2.1		0	1.0	
Marital status	Single	90.6	94.9	0.400	94.4	93.3	0.833
	Married	6.3	3.9		3.7	5.3	
	Others	3.1	1.2		1.9	1.5	
Education	Middle school	3.2	2.1	0.892	3.7	4.2	0.181
	High school	52.4	54.5		54.7	63.8	
	College	34.2	34.2		34.6	20.5	
	Graduation	10.3	9.2		7.0	11.5	
Socioeconomic Status^¥^	A	33.9	30.5	0.253	24.7	23.0	0.496
	B	51.2	57.7		62.8	58.5	
	C	14.9	10.7		11.9	16.5	
	D/E	0	1.1		0.6	2.0	
Illicit drug use inside nightclub	No	66.4	57.8	0.120	81.4	67.6	0.052
	Yes	33.6	42.2		18.6	32.4	

^¥^ Socioeconomic status classification by ABEP [37].

* Rao-Scott chi-square test [40].

^**§**^ BrAC—Breath alcohol concentration.

**Table 3:** Weighted distribution of risk behaviors and alcohol effects after leaving a nightclub, according to sex and BrAC measured at nightclub exits among patrons in São Paulo, Brazil–“*Balada com Ciência*” portal survey (N = 664 drinkers).

			MEN			WOMEN		
			BrAC^§^ 0.01–0.37 mg/L	BrAC≥0.38 mg/L		BrAC 0.01–0.37 mg/L	BrAC≥0.38 mg/L	
			(no binge drinking)	(binge drinking)		(no binge drinking)	(binge drinking)	
Variables		Categories	(N = 197)	(N = 205)	p-value*	(N = 124)	(N = 116)	p-value*
**Risk behavior**	Illicit drug use after leaving	Yes	9.9	20.7	<0.001	8.5	11.5	0.496
	Drink and drive	Yes	20.6	14.9	0.105	10.7	8.8	0.602
	New alcohol use	Yes	11.8	10.8	0.718	3.4	17.7	0.005
	Violent behavior	Yes	3.6	5.3	0.442	3.7	5.9	0.449
	Accidents	Yes	0.9	1.1	0.853	1.1	0.5	0.507
	Sexual risk behavior	Yes	9.4	13.2	0.268	7.6	5.0	0.285
	Policy involvement	Yes	0.3	0.3	0.971	1.1	0.5	0.507
**Alcohol effects**	Physical complications	Yes	4.3	14.1	<0.001	9.5	16.6	0.122
	Blackout	Yes	3.1	21.3	<0.001	4.7	20.5	<0.001

* Rao-Scott chi-square test [40].

^**§**^ BrAC—Breath alcohol concentration.

The associations between each risk behavior practiced after leaving the nightclub by sex and according to the BrAC levels measured at the exit of the nightclub are presented in Tables 4 and 5. For both sexes, a BrAC ≥0.38 mg/L was positively associated with risk behaviors and/or alcohol effects, after adjusting for potentially confounding variables. Considering risk behaviors, the practice of binge drinking increased the behavior of illicit drug use after leaving the nightclub by 2.54 times [95% CI: 1.26–5.09] among men and increased the risk of a new episode of alcohol use by 5.80 times [95% CI: 1.50–22.44] among women. Physical complications and blackouts were more likely to be reported among men and women identified with a binge drinking measure. Among men, binge drinking increased the occurrence of physical complications by 3.51 times [95% CI: 1.96–6.30] and the occurrence of "blackout" by 8.92 times [95% CI: 3.83–20.80] in the adjusted models. For women, binge drinking increased the occurrence of physical complications by 2.40 times [95% CI: 1.20–4.70] and the occurrence of "blackout" by 5.31 times [95% CI: 1.68–16.84] in the adjusted models.

**Table 4:** Association between risk behaviors and alcohol effects after leaving a nightclub and binge drinking BrAC measure at nightclub exits among male patrons in São Paulo, Brazil—*Balada com Ciência* portal survey (N = 402 men drinkers).

		MEN BrAC≥0.38 mg/L^§^ **			
	Variables*	UnOR (95% CI)	p-value	^$^AdOR (95% CI)	p-value
**Risk behavior after leaving the nightclub**	Illicit drug use after leaving	2.36(1.46–3.82)	0.001	2.54(1.26–5.09)	**0.010**
	Drink and drive	0.68(0.42–1.09)	0.107	0.66(0.39–1.13)	0.131
	New alcohol use	0.90(0.51–1.60)	0.711	0.83(0.47–1.50)	0.535
	Violent behavior	1.50(0.51–4.40)	0.445	1.52(0.51–4.54)	0.442
	Accidents	1.21(0.18–8.30)	0.836	1.13(0.16–7.84)	0.898
	Sexual risk behavior	1.47(0.73–3.00)	0.270	1.50(0.76–2.97)	0.237
	Policy involvement	0.94(0.50–17.65)	0.971	0.97(0.05–18.9)	0.983
**Alcohol effects**	Physical complications	3.65(20.7–6.43)	<0.001	3.51(1.96–6.30)	**<0.001**
	Blackout	8.50(3.74–19.16)	<0.001	8.92(3.83–20.80)	**<0.001**

* Risk behaviors/alcohol effect were the dependent variables.

^**§**^ BrAC—Breath alcohol concentration.

** BrAC was the independent variable, considering no binge drinking as the reference (reference category = BrAC 0.01–0.37mg/L).

^$^ Final logistic model was adjusted by age, marital status, education, socioeconomic status and drug use inside the nightclub and the negative variables was references (e.g., no drink and drive, no new alcohol use, no accidents).

**Table 5:** Association between risk behaviors and alcohol effects after leaving a nightclub and binge drinking BrAC measures at nightclub exits among female patrons in São Paulo, Brazil—*Balada com Ciência* portal survey (N = 242 women drinkers).

		WOMEN BrAC≥0.38 mg/L^§^ **			
	Variables*	UnOR (95% CI)	p-value	^$^AdOR (95% CI)	p-value
**Risk behavior after leaving the nightclub**	Illicit drug use after leaving	1.39(0.52–3.74)	0.498	0.64(0.18–2.44)	0.535
	Drink and drive	0.80(0.33–1.90)	0.603	0.84(0.29–2.40)	0.738
	New alcohol use	5.86(1.58–21.8)	0.010	5.80(1.50–22.44)	**0.013**
	Violent behavior	1.60(0.45–5.73)	0.453	1.29(0.35–4.63)	0.691
	Accidents	0.40(0.02–7.08)	0.521	0.36(0.03–4.14)	0.399
	Sexual risk behavior	0.63(0.26–1.51)	0.289	0.61(0.28–1.35)	0.215
	Policy involvement	0.40(0.03–7.10)	0.521	0.36(0.03–4.14)	0.399
**Alcohol effects**	Physical complications	1.89 (0.82–4.33)	0.126	2.40(1.20–4.70)	**0.015**
	Blackout	5.25(1.94–14.22)	0.002	5.31(1.68–16.84)	**0.006**

* Risk behaviors/alcohol effect were the dependent variables.

^**§**^ BrAC—Breath alcohol concentration.

** BrAC was the independent variable, considering no binge drinking as the reference (reference category = BrAC 0.01–0.37mg/L).

^$^ Final logistic model was adjusted by age, marital status, education, socioeconomic status and drug use inside the nightclub and the negative variables was references (e.g., no drink and drive, no new alcohol use, no accidents).

## Discussion

This study has evaluated the possible effects of a BrAC equivalent to binge drinking practices on risk behaviors among men and women at nightclub exits in Sao Paulo, Brazil. The results suggested that the prevalence of the practice of binge drinking was the same for both sexes. For men and women, the most prevalent risk behaviors practiced after leaving a nightclub were drinking and driving, the use of illicit drugs and risky sexual behavior. Concerning the harmful effects of alcohol, blackout episodes after leaving the nightclub were reported for nearly 10% of the patrons of these venues. In addition, men and women who practiced binge drinking were more likely to report episodes of blackout and physical complications of alcohol consumption than drinkers who left the establishments with lower dosages. Gender differences were observed regarding risk behaviors according to BrAC. Among women, the practice of a new episode of alcohol consumption after leaving the nightclub was significantly higher among patrons with a binge drinking pattern. Among men, the same was true for an increase in the use of illicit drugs after leaving the venue. In both sexes, the behaviors were more prevalent among patrons whose BrAC was equivalent to binge drinking compared with patrons whose BrAC was lower than that of binge drinking practices.

It is noteworthy that studies at nightclubs usually give attention to events occurring within the venue and their association with reported or measured alcohol intoxication [42, 43]. In the present study, we pushed forward to understand what happens after a patron leaves a nightclub; in other words, we investigated the most common effects of alcohol and risk behaviors to which they are exposed and their associations with the patrons’ BrAC when they left the venue.

The prevalence of patrons detected with a binge drinking BrAC in Sao Paulo nightclubs was lower than that found in a study conducted in bars and nightclubs of four European cities [33], in which 50.4% of respondents had engaged in binge drinking. It was also lower than the 35.5% prevalence of binge drinking identified among North-Americans approached on the Mexican-US border when returning from bars and nightclubs in the city of Tijuana [44]. In addition, the prevalence of 22% among women and 29% among men for the practice of binge drinking on the day of the nightclub survey was similar to that found for the Brazilian general population when considering a full year interval to measure binge drinking in the past year [45]. However, there has been no previous study on Brazilian drinking environments that allows a proper comparison for trends in prevalence.

Drinking and driving appeared to be the most common risk behavior among patrons after leaving the nightclub. Nevertheless, among this population, increased alcohol breath concentration did not appear to influence this behavior; thus, for both sexes, there was no statistically significant association between drinking and driving and the practice of binge drinking. However, Voas et al. [46] suggested that the efforts of public policies to reduce the pattern of drinking and driving between patrons at drinking locations have shown effects because over time, there appears to be a reduction in the number of drivers with high alcohol blood concentration returning from drinking locations. The present study highlights that the rate of individuals who drink and drive (27.9% of men and 20.4% women) was much higher than the 4.2% found in a representative sample of a population of drivers from Brazilian roads [47]. However, it should be noted that Brazil has one of the most stringent laws for alcohol consumption and traffic. This law, known in Brazil as the "dry law", indicates that no alcohol blood/breath concentration is accepted among drivers, and the penalty for a BrAC >0.01 mg/L ranges from fines to car seizure and arrests (Law No. 12,760 / 2012). However, we have noted that a large portion of the patrons do not felt intimidated by this law, which suggests the need for law enforcement and more sobriety checkpoints.

The use of illicit drugs following the departure of nightclubs, which was already considerably higher than the past month illicit drug use among the Brazilian general population (4.5%) [48], was increased among men with a binge drinking BrAC. However, this percentage was much lower than that found in a study in San Francisco (USA), in which it was estimated that 30% of nightclub attendees had used illicit substances [46]. Nevertheless, we highlight here a greater exposure to the risks of legal, psychological and physical consequences of drug use associated with alcohol use by characterizing polydrug use [49] practiced by these respondents.

Among women, a notable risk behavior increased by binge drinking was the new use of alcohol, in other words, additional drinking after leaving the nightclub. This behavior requires attention because it may suggest a potential alcohol craving for higher alcohol doses induced by the alcohol intoxication, suggesting that one’s own alcohol intoxication prevents adequate control of the amount of alcohol ingested [50]. Thus, in this scenario, women who were already intoxicated, particularly those who should not continue consuming alcohol that night, were the ones most likely to report a new alcohol use. This behavior increases the risk inherent to the alcohol intoxication, which can lead to an alcoholic coma or sexual abuse, as described by Testa et al. [51].

There were also clear deleterious effects of alcohol on the body in approximately 10% of the patrons who reported blackouts and/or physical complications because of alcohol consumption. Again, we face the question of the limits of consumption. It was expected that binge practices would be associated with an increase of these physical effects; however, surprisingly, binge drinking increased the occurrence of these effects by 9 times in men and 5 times in women. Blackouts are one of the most complex effects of alcohol intoxication and are responsible for psychological stress among patrons because this exposes them to risk behaviors that they cannot even report because of the memory loss associated with the blackout [52]. Considering this scenario, all the behaviors reported the next day by these patrons may be underestimated because they do not remember parts of the end of the night.

The scientific literature is scarce regarding the population prevalence of alcohol induced harm in drinking environments. In this sense, this study fills an important gap in the field of individual and public health by identifying the possible role of binge drinking. However, regardless of binge drinking, sexual risk behavior, which is more prevalent among men, draws attention for its potential life-long effects. In Brazil, according to recent governmental data [53], the recrudescence of the HIV epidemic is concentrated among young adults aged 20–25 years; this is coincidently the age category investigated in this survey. This phenomenon indicates the need for tailored campaigns to raise awareness of contagion risks for several STDs in these establishments because of the behavior after leaving the venue and the influence of alcohol on decision making.

The findings presented here are interesting not only in what was found but also in what was not found. Explicitly, many risk behaviors that have previously been identified as associated with binge drinking were not identified in this study in a drinking environment, such as sexual risk behaviors and violent behaviors [27, 54]. However, the binge drinking definition of 5/4 (men/women) doses was established according to findings that after the consumption of this amount or more, individuals are at a greater risk for exhibiting serious alcohol-related problems, such as fights, trouble with police, injuries, and drunk driving, among other subsequent negative health and social consequences [5]. Therefore, this study suggests that ethnicity and sociocultural aspects may influence the binge cutoff measure for the Brazilian population because the majority of the risk behaviors were not associated with the National Institute on Alcohol Abuse and Alcoholism (NIAAA) binge drinking measure of 0.08% BAC (or a BrAC ≥ 0.38 mg/L) [7, 39].

Although this is an innovative study, some limitations are present. The first is the sample loss to follow-up during the different phases of the study. Of the 1832 patrons whose BrAC was measured at the exit of the nightclubs, only 1222 of them answered the online questionnaire 24 hours later that reported their behavior practiced after leaving the venue. Also, the exclusion of highly intoxicated patrons at the exit (n = 67 cases) can probably have underestimate some risky behavior prevalence. In addition, the small number of cases in some response categories, such as "accidents" and "policy involvement” (problems with the police) were difficult to analyze in the logistic regression model. Although an online, anonymous questionnaire increases the validity of the reporting of illegal activities or risk behaviors, it is noted here that the alcoholic blackout could have compromised the validity of the response of the patrons who experienced it.

## Conclusions

We note that exposure to risk behaviors among patrons is not limited to events within the venue but also to events occurring after the departure of these establishments and appear to be associated with alcoholic consumption. These results suggest that public policy should provide more efforts at reducing alcoholic harm among nightclubs patrons, taking into account that the risk episodes may occur just after leaving the venue. One of the initial measures to guarantee a lower BrAC at nightclub exits would be nightclub staff training in responsible beverage service (RBS) and future legislation to prevent alcohol sales to drunk individuals, as evidenced by the study of Hughes et al. [55]. However, it is worth noting that the alcohol consumption market in Brazil is unregulated [56], and few or no actions to regulate the physical availability of alcohol [57] have been tested in Brazil. Therefore, it is suggested that the results of this study can guide international effective alcohol public policies aimed at the protection of nightclub patrons by using a triad of education, regulation and effective enforcement, which will allow observable changes in future generations.

## Supporting Information

S1 File Survey dataset. Dataset of the variables used in this manuscript.
(XLSX)
